# The degree of local inflammatory response after colonic resection depends on the surgical approach: an observational study in 61 patients

**DOI:** 10.1186/s12893-015-0097-y

**Published:** 2015-10-06

**Authors:** Torben Glatz, Ann-Kathrin Lederer, Birte Kulemann, Gabriel Seifert, Philipp Anton Holzner, Ulrich Theodor Hopt, Jens Hoeppner, Goran Marjanovic

**Affiliations:** Department of General and Visceral Surgery, Albert Ludwigs University of Freiburg, Freiburg im Breisgau, Germany

**Keywords:** Laparoscopic surgery, Laparoscopically-assisted surgery, Peritoneal inflammation, Cytokines, Wound healing

## Abstract

**Background:**

Clinical data indicate that laparoscopic surgery reduces postoperative inflammatory response and benefits patient recovery. Little is known about the mechanisms involved in reduced systemic and local inflammation and the contribution of reduced trauma to the abdominal wall and the parietal peritoneum.

**Methods:**

Included were 61 patients, who underwent elective colorectal resection without intraabdominal complications; 17 received a completely laparoscopic, 13 a laparoscopically- assisted procedure and 31 open surgery. Local inflammatory response was quantified by measurement of intraperitoneal leukocytes and IL-6 levels during the first 4 days after surgery.

**Results:**

There was no statistical difference between the groups in systemic inflammatory parameters and intraperitoneal leukocytes. Intraperitoneal interleukin-6 was significantly lower in the laparoscopic group than in the laparoscopically-assisted and open group on postoperative day 1 (26.16 versus 43.25 versus 40.83 ng/ml; *p* = 0.001). No difference between the groups was recorded on POD 2–4. Intraperitoneal interleukin-6 showed a correlation with duration of hospital stay on POD 1 (0.233, *p* = 0.036), but not on POD 2–4.

Patients who developed a surgical wound infection showed higher levels of intraperitoneal interleukin-6 on postoperative day 2–4 (POD 2: 42.56 versus 30.02 ng/ml, *p* = 0.03), POD 3: 36.52 versus 23.62 ng/ml, *p* = 0.06 and POD 4: 34.43 versus 19.99 ng/ml, *p* = 0.046). Extraabdominal infections had no impact.

**Conclusion:**

The analysis shows an attenuated intraperitoneal inflammatory response on POD 1 in completely laparoscopically-operated patients, associated with a quicker recovery. This effect cannot be observed in patients, who underwent a laparoscopically-assisted or open procedure. Factors inflicting additional trauma to the abdominal wall and parietal peritoneum promote the intraperitoneal inflammation process.

## Background

Major abdominal surgery and laparotomy causes a release of local and systemic cytokines, inducing a systemic inflammatory response syndrome [1–3]. Local peritoneal inflammation is thought to play a role in patient recovery and in development of perioperative complications [4–7]. Laparoscopic surgery has been shown to attenuate both local and systemic inflammatory response [8–13].

Today, minimally-invasive bowel resection has been established as a standard procedure in most hospitals. Retrospective analyses indicate a beneficial effect of laparoscopically- performed surgery on patient recovery and even on the occurrence of serious complications like anastomotic leak and on patient survival [14–17].

In an attempt to quantify the systemic and local impact of laparoscopic surgery on the inflammatory response, systemic and intraperitoneal (ip) interleukin-6 (IL-6) has been established as a sensitive marker of inflammation on postoperative day 1 after surgery. Low ip levels of IL-6 are associated with faster recovery and earlier hospital discharge [13, 18]. Several studies show lower levels of systemic IL-6 on day 1 after laparoscopic surgery [12], while only one study exists illustrating the effect of laparoscopic surgery on ip IL-6 [13]. All others studies have failed to establish a difference in intraperitoneal cytokines between laparoscopic and conventional surgery [19–21]. Most of these studies did not include completely laparoscopic operations, but only laparoscopically-assisted procedures with laparotomy after colonic mobilization, which might induce an increased local inflammatory response compared to a completely laparoscopic procedure. The aim of this study is to determine whether postoperative intraperitoneal inflammation depends on the surgical technique and the trauma inflicted on the abdominal wall by quantifying local inflammatory response via ip IL-6 levels during the first days after colorectal surgery in patients undergoing a completely laparoscopic, a laparoscopically-assisted or an open procedure.

## Methods

### Patients and operative procedure

The observational study includes 61 consecutively operated patients, who underwent elective colorectal resection from January to December 2013 at our institution. Only patients without major intraabdominal complications were included. Informed consent was obtained from all patients before operation and inclusion in the study. Institutional Review Board approval was granted for the sample collection and evaluation of patient demographics (EK-F: 345/12) by the Medical Ethics Committee of the University of Freiburg, the study was carried out according to the Helsinki Declaration. Data were collected with regard to patient demographics, details of disease, operative procedure and extraabdominal complications. Complications were defined by standardized definitions and graded according to the Clavien-Dindo classification [22, 23].

Procedures included in the study were left-sided colon resections (sigmoid resection [*n* = 21], left hemicolectomy [*n* = 19]) and anterior rectum resection (*n* = 21). All operations were performed by or under supervision of experienced colorectal surgeons. The surgical approach (laparoscopic, laparoscopically-assisted or open) was chosen by the operating surgeon. Patients were not randomized to the groups. The patients were retrospectively assigned to the group according to the operation technique. Four operations were converted from laparoscopic to open and were assigned to the open group. The laparoscopically-assisted procedure was defined as a laparoscopic mobilization of the colon and fashioning of the anastomosis through a small lower medial laparotomy (6–12 cm), while the completely laparoscopic operation was characterized by laparoscopic fashioning of the anastomosis and removal of the resected bowel through a suprapubic mini-laparotomy (3–5 cm) after complete laparoscopic mobilization. Our definitions are concordant with the literature [24, 25].

Postoperatively, patients received standardized pain medication (metamizole, oxycodone and piritramide). Anti-inflammatory drugs were omitted whenever possible.

### Sample collection and storage

Patients included in the study had a 10 mm Intersil® Silicone X-Ray Capillary Drain (Mikrotek® medical, Mosta, MT) inserted into the Douglas cavity during surgery. The intraperitoneal fluid was collected in a collecting bag (Urine bag, Asis Bonz®, Herrenberg, DE). The drain was removed on postoperative day 4 after collection of samples.

Drainage clearance was performed daily at 6 am. Two to four hours later, fresh drainage fluid was collected on POD 1–4 and venous blood samples were taken on POD 1, 3, and 5. All samples were sent immediately to the Freiburg university hospital laboratory for further analyses.

### Parameter analyses

Analyzed were serum leukocytes on POD 1, 3 and 5 and serum c-reactive protein (CRP) on POD 3 and 5 as part of the routine follow-up. Intraperitoneal leukocytes and IL-6 were measured on POD 1–4. In 6 patients, measurement of intraperitoneal leukocytes was not possible due to lack of material. On POD 4, the drain had already been removed in 9 patients, thus no material was taken for analyses. Parameter analyses were performed with the modular analyzer Cobas® 8000 (Roche® Diagnostics, Rotkreuz, Risch, CH) for IL-6 and CRP and the XN-9000® (Sysmex® Corporation, Kobe, JP) for leukocytes.

### Statistical analysis

Results are expressed as mean ± SD and medians with range, as appropriate. The primary outcome parameter was ip IL-6, the secondary outcome measures were ip leukocytes, systemic leukocytes, CRP and patient demographics. Differences between categorical variables were evaluated by Fisher’s exact test. Differences between continuous variables were measured using the Kruskal-Wallis test or Mann–Whitney-U-test, as appropriate. SPSS for Windows^™^ was used for statistical analysis (SPSS, Chicago, IL, USA). A Spearman’s correlation coefficient was employed to express the correlations. *P* < 0.05 was considered significant.

## Results

### Patients

Among the 61 patients, 17 were operated completely laparoscopically, 13 laparoscopically- assisted with additional laparotomy and 31 underwent open surgery. Twenty-six patients were male and 35 female, the mean age was 56.7 years (±16.4). Thirty-one patients were operated with an underlying benign disease, 30 had a malignant disease. Mean operating time was 193 min (Table 1). There were no significant differences in sex (*p* = 0.10), age (*p* = 0.29) or performed procedure (*p* = 0.24) between the groups, though the majority of patients in the laparoscopic group were women (76 %). There were more malignancies in the open and laparascopically-assisted groups than in the laparoscopic group (*p* = 0.04).

**Table 1:** Patient demographics and treatment specifications

	Total (*n* = 61)
Age (years, mean ± SD)	56.7 ± 16.4
Sex	
Male (*n*)	43 % (26)
Female (*n*)	57 % (35)
Disease (*n*)	
Benign	51 % (31)
Malignant	49 % (30)
Operation (*n*)	
Left-sided colon resection	66 % (40)
Anterior rectum resection	34 % (21)
Operating time (min, mean ± SD)	193 ± 72
Extrabdominal complications (grade)	26 % (16)^a^
Wound infection (II-IIIa)	14 % (8)
Urinary-tract-infection (II)	10 % (6)
Pneumonia (II)	4 % (2)
Other (II-IIIa)	4 % (2)
Hospital stay [days, median (range)]	12 (6–20)

^a^2 patients had more than one complication

### Systemic inflammatory parameters and intraperitoneal leukocytes

Systemic leukocytes were mildly elevated on POD 1 (11.71 thsd/μl) and returned to normal values on POD 3 and 5 (Table 2). There was no statistical difference among the three groups. Serum CRP-levels tended to be higher in the open compared to the completely laparoscopically-operated group on POD 3 (119 versus 82 mg/l) without being significant (*p* = 0.22). There was no difference among the groups on POD 5 (Table 2).

**Table 2:** Systemic and local inflammatory parameters after open and laparoscopic surgery

	POD	Total (*n* = 61)	Laparoscopic (*n* = 17)	Lap.-assisted (*n* = 13)	Open (*n* = 31)	P^a^
Leukocytes (serum)	**1**	11.71 ± 4.40	10.91 ± 4.35	10.75 ± 3.19	12.56 ± 4.81	0.39
	**3**	8.41 ± 3.31	8.07 ± 4.13	8.40 ± 3.21	8.61 ± 2.94	0.70
Thsd/μl	**5**	7.50 ± 2.93	7.76 ± 4.11	7.52 ± 2.42	7.35 ± 2.40	0.99
CRP	**3**	104 ± 67	82 ± 65	100 ± 58	119 ± 69	0.22
mg/l	**5**	69 ± 57	75 ± 70	59 ± 48	70 ± 53	0.55
Leukocytes ip	**1**	10.67 ± 12.93	11.16 ± 14.48	9.23 ± 6.18	10.89 ± 14.02	0.83
Thsd/μl	**2**	8.39 ± 13.25	11.52 ± 18.02	3.02 ± 2.27	8.41 ± 11.84	0.38
	**3**	9.73 ± 20.34	18.69 ± 32.64	6.17 ± 11.25	5.57 ± 8.99	0.92
	**4**	6.01 ± 13.93	14.43 ± 25.89	3.5 ± 6.24	3.31 ± 5.28	0.41
IL-6 ip	**1**	**37.26 ± 15.30**	**26.16 ± 17.53**	**43.25 ± 10.47**	**40.83 ± 12.74**	**0.004**
ng/ml	**2**	31.66 ± 16.59	27.56 ± 17.43	35.34 ± 14.51	32.37 ± 16.97	0.30
	**3**	25.31 ± 16.78	22.38 ± 16.44	29.26 ± 18.17	25.25 ± 16.58	0.59
	**4**	21.93 ± 16.91	21.50 ± 20.18	21.50 ± 16.91	22.31 ± 16.19	0.93

Results are presented as mean ± SD in ng/ml. Significant results are bold

^a^Kruskal-Wallis test

Intraperitoneal leukocytes were very heterogeneously distributed with high standard deviation and no significant difference between the groups (Table 2).

### Patient demographics, surgical technique and intraperitoneal IL-6

**Fig. 1:**
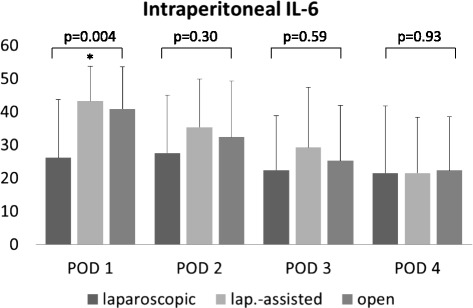
Postoperative release of intraperitoneal IL-6. Intraperitoneal levels of IL-6 are lower in patients who underwent a completely laparoscopic procedure on POD 1. Values are expressed as ng/ml, mean ± SEM

Mean total values of ip IL-6 were very high on POD 1 (37.26 ng/ml) and were halved by POD 4 (Table 2). Levels were significantly lower in the completely laparoscopically-operated group on POD 1 compared to the laparoscopically-assisted and the open group (26.16 versus 43.25 versus 40.83 ng/ml; *p* = 0.004; Fig. 1).

Age, type of operation (left-sided colon resection versus anterior rectum resection) and type of disease (malignant versus benign) had no impact on the concentration of ip IL-6. Female patients showed consistently lower levels than male patients on all postoperative days (POD 1: 42.18 versus 33.60 ng/ml, *p* = 0.007; POD 2: 37.11 versus 27.62 ng/ml, *p* = 0.03; POD 3: 32.28 versus 20.13 ng/ml, *p* = 0.004; POD 4: 27.07 versus 17.52 ng/ml, *p* = 0.04).

Separate analysis of the female group (*n* = 34) showed consistent results with the overall collective: Female patients who underwent a completely laparoscopic procedure had lower levels of ip IL-6 than those after laparoscopically-assisted or the open procedure on POD 1 (female patients: 23.92 versus 41.29 versus 38.74 ng/ml, *p* = 0.03).

### Perioperative complications and intraperitoneal IL-6

The study only includes patients without intraabdominal complications (anastomotic leak, abscess). There were no mortalities. Twenty-six percent of the patients had a perioperative complication (the most common being wound infection and urinary tract infection), one patient had a deep vein thrombosis and was treated with i.v. heparin, another suffered from NSTEMI and underwent percutaneous coronary intervention. The number of complications did not differ among the groups (*p* = 0.40).

**Fig. 2:**
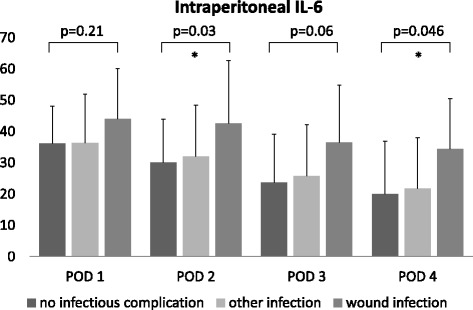
Impact of perioperative complications on intraperitoneal IL-6 release. Patients with surgical wound infections have higher levels of intraperitoneal IL-6, while other infections have no impact. Values are expressed as ng/ml, mean ± SEM

Patients who developed wound infection showed a higher IL-6 concentration on POD 2 (42.56 versus 30.02 ng/ml, *p* = 0.03), POD 3 (36.52 versus 23.62 ng/ml, *p* = 0.06) and POD 4 (34.43 versus 19.99 ng/ml, *p* = 0,046), while pulmonary and urinary tract infections had no impact on ip IL-6-levels (Fig. 2).

### Hospital stay and Intraperitoneal IL-6

Hospital stay was significantly lower in the laparoscopic and the laparoscopically-assisted than in the open group (*p* = 0.01). There was no difference between the two laparoscopic groups (*p* = 0.77, Table 1). To analyze a possible correlation between ip IL-6 levels and the duration of hospital stay, we calculated a Spearman’s correlation coefficient for these variables. A positive correlation was found for ip IL-6 concentration on POD 1 (0.233; *p* = 0.04), but not on POD 2 (0.058, *p* = 0.66), POD 3 (0.090, *p* = 0.49 or POD 4 ( −0.062, 0.66).

## Discussion

Quantification of local and systemic inflammatory response has shown a decreased inflammatory response after laparoscopic compared to open surgery [8–13]. Intraperitoneal IL-6 is a promising parameter of intraperitoneal inflammatory response after different kinds of abdominal surgery. Low intraperitoneal levels of IL-6 during the first 24 h after colonic surgery correlate with faster recovery and earlier hospital discharge [13] while high levels seem to favour the development of postoperative fatigue [18].

IL-6 has primarily been studied during the initial 24 h after surgery. We present the first study analyzing the physiological course of ip IL-6 levels over 4 days after colonic surgery.

In this study, patients were not randomized to the groups and not matched for type of operation and disease, sex and age to ensure best possible care for every patient. While type of disease had no impact on ip IL-6 levels, surprisingly female patients had consistently lower levels of ip IL-6, but separate analysis of the female group confirmed our findings: Lower levels of ip IL-6 after completely laparoscopic surgery on POD 1. Deviation in cytokine response under different circumstances among men and woman has been reported before in different medical fields [26–28], but reasons remain unexplained.

Decreased levels of ip IL-6 on POD 1 after laparoscopic surgery were shown for the first time in 2012 [13]. In addition, we were able to show that this effect does not apply to patients who underwent a laparoscopically-assisted procedure. A few earlier analyses studied the effect of laparoscopic surgery on cytokine release. These studies were able to show a diminished systemic release of IL-6 after laparoscopic surgery, but failed to show a difference in ip IL-6 levels [19–21]. Study design was compromised by a small number of patients and also included mainly patients who underwent a laparoscopically-assisted procedure and not a completely laparoscopic operation. Our data indicate that the local inflammatory response after laparoscopically-assisted surgery compares to that following open surgery. Thus, additional laparotomy and therefore increased trauma to the abdominal wall and the parietal peritoneum seems to promote intraperitoneal inflammation.

The factors actually responsible for the clinically-observed beneficial effects of laparoscopic surgery are not well known [29, 30]. Some studies show that the effects of the pneumoperitoneum on peritoneum and wound healing compare to those of a laparotomy [31–33]. In our setting, however, local inflammation depended partly on the amount of surgical trauma inflicted to the abdominal wall. Until today, many surgeons have opted for a laparoscopically-assisted procedure because it has a flat learning curve, can be performed in a shorter period of time and offers a safe and easy way to fashion the anastomosis [24, 25]. Our data indicate, though, that the beneficial effects observed and proven for laparoscopic surgery regarding attenuated inflammatory response might not apply to laparoscopically-assisted surgery.

We and others [13] were able to show a correlation between ip IL-6 levels on POD 1 and patient recovery, supporting our hypothesis. However, in this study the difference in hospital stay between the laparoscopic and laparoscopically-assisted groups was not significant and the clinical relevance of an additional / longer abdominal wall incision seems to be rather low in this setting. The impact and significance of ip IL-6 levels at later time points after surgery remains unknown. Our data show no correlation between IL-6 levels and patient recovery on POD 2–4.

Intraperitoneal IL-6 has been proposed as a diagnostic tool for early detection of anastomotic leaks [34, 35]. In this study, patients who developed an anastomotic leak have higher levels of ip IL-6 on POD 1, 3 and 5 than patients with an uneventful postoperative course [35]. Patients with intraabdominal complications were excluded from this analysis, but the data show that surgical wound infections without involvement of the intraabdominal cavity stimulate release of ip IL-6. Levels were higher on POD 2–4, an effect possibly mediated by affection of the parietal peritoneum. Due to incomplete healing of the abdominal fascia, the infection can easily involve the parietal peritoneum. Besides the direct involvement of the peritoneum, we suspect involvement of other cytokines mediating intraperitoneal inflammation after injury of the abdominal wall. Intraperitoneal IL-6 was not affected by extraabdominal infections like pneumonia or urinary tract infection and therefore seems to be more specific than systemic inflammatory parameters.

Additionally, this finding contributes to our impression that trauma and inflammation of the abdominal wall and parietal peritoneum has an impact on the intraabdominal inflammation process. However the mechanisms by which extraperitoneal stimuli might or might not effect intraperitoneal inflammation are not completely understood.

In our study, analyses of ip leukocytes revealed no differences between different operating techniques. The significance of postoperative intraperitoneal leukocytes as a diagnostic marker for intraperitoneal inflammation or perioperative complications remains unknown [1, 2].

## Conclusions

We analyzed the physiological course of ip IL-6 over POD 1–4 in patients after colonic resection and were able to show an attenuated intraperitoneal inflammatory response on POD 1 only in completely laparoscopically-operated patients, while this effect could not be observed in patients who underwent a more traumatic laparoscopically-assisted or even open procedure. Thus, we conclude that the degree of intraperitoneal inflammation depends not only on the trauma inflicted on the bowel but also to a major part on the extent of trauma to the abdominal wall and parietal peritoneum. Even if the clinical significance of these data appears to be rather low, we provide further basic knowledge of the impact of laparoscopic techniques on the postoperative healing process which should be more precisely explored in future randomized trials.
